# Wellens syndrome in a young woman with coronary artery vasculitis

**DOI:** 10.1002/jgf2.467

**Published:** 2021-06-06

**Authors:** Ryohei Ono, Hidehisa Takahashi, Yasuhiko Hori, Kenichi Fukushima

**Affiliations:** ^1^ Department of Cardiovascular Medicine Chiba University Graduate School of Medicine Chiba Japan; ^2^ Department of Cardiology Matsudo City General Hospital Matsudo Japan

**Keywords:** computed tomography, coronary artery vasculitis, kawasaki disease

## Abstract

We report the case of a 30‐year‐old woman who was referred to our hospital with chest pain. An electrocardiogram showed biphasic T‐wave inversions in leads V2–4 compared with that done 2 years ago, suggesting Wellens syndrome, and an emergent coronary angiography revealed significant stenosis of the proximal left anterior descending artery due to coronary artery vasculitis. Although acute coronary syndrome in the young is very rare, coronary artery vasculitis should be considered as a possible etiology, especially in young women with chest pain.

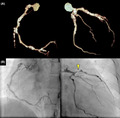

A 30‐year‐old woman presented with an acute onset of chest pain lasting 15 min, prior to visiting our hospital. While she was walking, squeezing chest pain and nausea suddenly developed. The pain did not radiate or worsen with respiration. She was a nonsmoker, and her family history was unremarkable.

She had no history of Kawasaki disease in her childhood but had a 1‐month history of fever of unknown origin when she was 18 years old. At that time, she did not visit a hospital and the fever spontaneously resolved without any treatment. When she was 28 years old, she experienced a 1‐week history of intermittent fever and visited our hospital. The fever already subsided at visit, and laboratory findings were unremarkable. Whole body computed tomography (CT) revealed no cause of fever but severe calcifications of coronary vessels. Coronary CT angiography revealed diffuse calcifications of the coronary vessels (Figure 1A). Since she had the history of unknown cause of fever 10 years prior, positron emission tomography (PET) CT was performed, but it showed no evidence of active vasculitis. Other possible diagnoses, including infections, connective tissue diseases, and IgG4‐related disease, were negative; thus, the patient was diagnosed she had had coronary artery vasculitis. Since she did not have any symptoms, she had been followed up annually without any medication.

**FIGURE 1:**
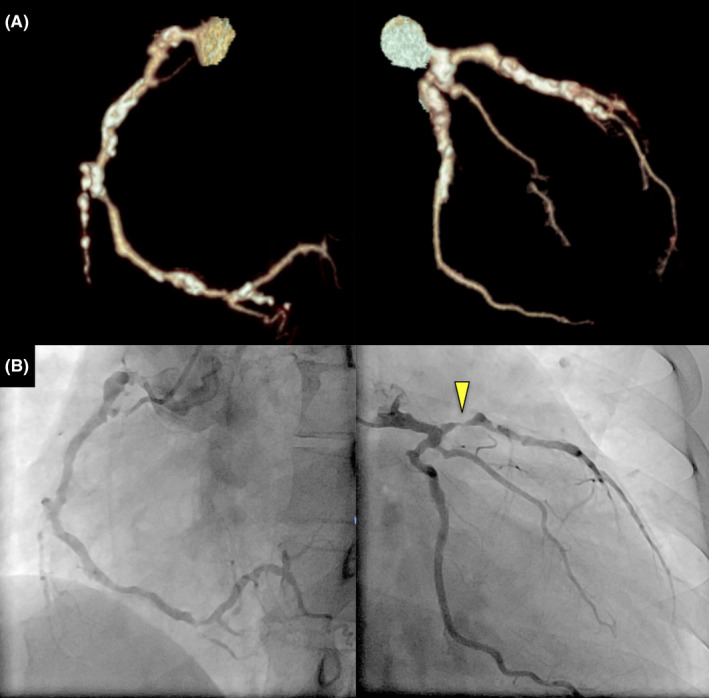
(A) Coronary computed tomography angiography 2 years ago showing diffuse calcifications in the coronary vessels. (B) Present coronary angiography revealing significant stenosis of the proximal left anterior descending artery

On arrival, she was afebrile without chest pain. Her heart rate was 84 beats per minute, and her blood pressure was 128/78 mm Hg. Physical examination was unremarkable. An electrocardiogram showed biphasic T‐wave inversions in leads V2–4 compared with that done 2 years ago, suggesting Wellens syndrome (Figure 2A,B). Laboratory findings revealed no elevation of creatine kinase, creatine kinase‐MB, low‐density lipoprotein cholesterol (60 mg/dl; normal range 70–139), and hemoglobin A1c (5.1%; 4.2–6.2); however, troponin T was slightly elevated (16.6 ng/L; <14). An emergent coronary angiography revealed significant stenosis of the proximal left anterior descending artery (Figure 1B). Percutaneous coronary intervention was subsequently performed, and the chest pain resolved after the procedure. She was treated with antiplatelet agents and statin, and the patient had not had any symptoms of fever or chest pain at 1‐year follow‐up.

**FIGURE 2:**
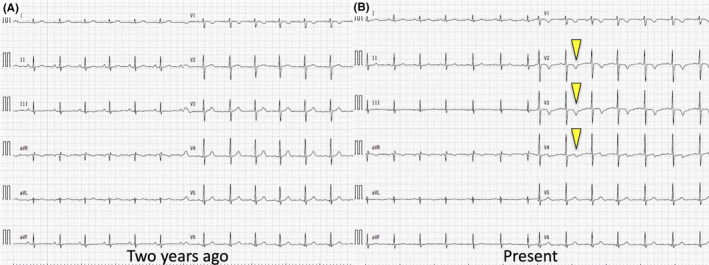
(A) An electrocardiogram 2 years ago. (B) A present electrocardiogram demonstrating biphasic T‐wave inversions in leads V2–4

The vasculitis of the coronary vessels could be caused by Kawasaki disease, Takayasu arteritis, giant‐cell vasculitis, Behcet's disease, polyarteritis nodosa, IgG4‐associated vasculitis, and syphilitic aortitis.^1^ This case did not meet any criteria of them specifically; thus, a diagnosis of idiopathic coronary artery vasculitis was made.

Wellens syndrome is defined by deeply inverted or biphasic T waves in V2–3 and is associated with previous angina, normal to slightly elevated cardiac markers.^2^ Wellens syndrome is typically suggestive of severe proximal stenosis of the left anterior descending artery and is highly specific for an imminent anterior wall myocardial infarction.^3^, ^4^ Its association with coronary artery vasculitis has not been reported previously. Although acute coronary syndrome in the young is very rare, coronary artery vasculitis should be considered as a possible etiology, especially in young women with chest pain.^5^

## CONFLICT OF INTEREST

The authors have stated explicitly that there are no conflicts of interest in connection with this article.

## INFORMED CONSENT

We have obtained the consent of the patient for publication.
